# Psychological, social, and environmental factors to meeting physical activity recommendations among Japanese adults

**DOI:** 10.1186/1479-5868-6-60

**Published:** 2009-08-28

**Authors:** Ai Shibata, Koichiro Oka, Kazuhiro Harada, Yoshio Nakamura, Isao Muraoka

**Affiliations:** 1 Faculty of Sport Sciences, Waseda University, Saitama, Japan; 2 Graduate School of Sport Sciences, Waseda University, Saitama, Japan; 3 Research Fellow of the Japan Society for the Promotion of Science, Tokyo, Japan

## Abstract

**Background:**

Although the benefits of the recommended level of physical activity on reducing chronic diseases are well-established, most of the Japanese population is not sufficiently active. Thus, examining correlates is an important prerequisite for designing relevant polices and effective programs. The present study investigated psychological, social, and environmental factors associated with meeting physical activity recommendations among Japanese adults.

**Methods:**

Data were analyzed for 1,932 men and women (43.6 ± 13.0 years), who responded to an Internet-based cross-sectional survey. Self-reported measure of physical activity, psychological (self-efficacy, pros, and cons), social (social support, health professional advice), environmental (home fitness equipment, access to facilities, neighborhood safety, enjoyable scenery, frequently observing others exercising, residential area), and demographic (gender, age, marital status, educational level, household income level, employment status) variables were obtained. Based on the current national guidelines for exercise in Japan (23 METs·hour per week), respondents were divided into two categories–recommended and not recommended (insufficient and inactive)–according to their estimated weekly physical activity level. An adjusted logistic regression model was utilized.

**Results:**

When adjusting for all other variables, self-efficacy (men: OR = 2.13; 95% CI: 1.55–2.94, women: OR = 2.72; 95% CI: 1.82–4.08) and possessing home fitness equipment (men: OR = 1.55; 95% CI: 1.14–2.10, women: OR = 1.41; 95% CI: 1.01–1.99) for both genders, social support (OR = 1.44; 95% CI: 1.06–1.97) for men, and enjoyable scenery (OR = 1.60; 95% CI: 1.09–2.36) for women were positively associated with attaining the recommended level of physical activity. In women, cons (OR = 0.47; 95% CI: 0.33–0.67) and living in rural areas (OR = 0.50; 95% CI: 0.25–0.97) were negatively associated with meeting the physical activity recommendations.

**Conclusion:**

In the psychological, social, and environmental domains, significant correlates of attaining the recommended level of physical activity were observed. Men and women had different patterns of psychological, social, and environmental correlates. These findings suggest that an intervention design that accounts for those correlates may more effectively promote physical activity among Japanese adults.

## Background

Promoting engagement in regular physical activity is now a worldwide health priority for disease prevention policy. Regular physical activity decreases the risk of cardiovascular disease, stroke, hypertension, diabetes mellitus, obesity, some forms of cancer, osteoporosis, and other chronic conditions [1,2]. International public health guidelines for adults recommend obtaining at least 30 minutes of moderate-intensity physical activity five or more days per week [1,3-5]. However, 50% or more of the adult population in many industrialized countries is not active enough to receive the many health benefits [1,6,7]. According to the Japanese guidelines on health promotion and recommendations for physical activity and exercise, every adult should accumulate 23 METs·hour per week (METs·hour is a unit that expresses the quantity of physical activity; it is calculated by multiplying the intensity of the activity or MET by the duration of the activity) of physical activity at an intensity level of at least 3 METs to prevent chronic diseases and to obtain numerous health benefits [8]. However, previous cross-sectional studies, which utilized the International Physical Activity Questionnaire (IPAQ) to estimate the amount of physical activity, reported that less than 30% of the Japanese adult population achieves this [9,10]. Therefore, the need to identify effective population-based intervention strategies to promote physical activity is exceedingly evident.

A better understanding of the contributing factors that influence physical activity is critical in designing relevant policies and effective interventions because it allows researchers to focus on modifying these factors. Previously, numerous studies have focused exclusively on the psychological and social correlates of engaging in physical activity [11,12]. In the last decade, the ecological perspective–emphasizing that behavior is influenced by a complex interaction of multiple levels of factors (demographic, psychological, social, and environmental) and therefore that addressing variables at multiple levels is necessary to understand and change behavior–has been increasingly applied in identifying correlates of physical activity participation [13,14]. A large number of previous studies, which simultaneously included factors across multiple levels [15-24], reported that self-efficacy and several types of social support from family and friends were strong correlates of attaining the recommended physical activity level [15-18,21,23,24]. However, the strength and direction of the association of other psychosocial factors (intentions, perceived benefits, barriers to physical activity, etc.) and, in particular, environmental factors (access to facilities, neighborhood sidewalk and lighting, neighborhood safety from crime, aesthetics, etc.) with attaining the recommended level of physical activity continued to vary across previous multivariable studies [15-24].

Although multilevel approaches derived from the ecological framework have been recommended for investigating the correlates of physical activity participation, almost all the previous studies were conducted in the U.S. and Australia. Few studies examined the correlates of physical activity on the multilevel domains in the Japanese population [25]. Further, the environmental (higher residential density, lower crime rate, etc.) and cultural backgrounds in Japan are different from those in the U.S. and Australia [26,27]. Identifying these potential influential factors in the Japanese context would enable the design of more tailored intervention strategies to promote physical activity in Japan.

Finally, understanding the association of psychological, social, and environmental factors with engagement in physical activity in men and women is essential since the daily living environment differs for men and women, which could cause an uneven distribution of physical activity participation between the genders [10,11]. Previous research has observed different patterns of correlates for men and women [24,28-30]. Although factors such as social support for exercise, perceived barriers to physical activity, and seeing numerous people engaging in physical activity in the neighborhood were reported to be more influential for women [24,28-30], it is apparent that further studies are necessary. Therefore, the present study examined the psychological, social, and environmental factors associated with meeting physical activity recommendations among a sample of Japanese adult men and women.

## Methods

### Participants

The data sample for the current study consisted of 1,932 respondents to an Internet-based cross-sectional survey, which was conducted through a Japanese Internet research service organization that owns approximately 264,000 voluntarily registered samples across Japan. The organization had access to samples of detailed sociodemographic attributes and was able to target specific attributes according to the requirements of each survey. The set sample size and attributes in the current study were as follows: approximately 2,000 adults, 20–79 years of age, stratified by a distribution of Japanese national age, gender, and regional distributions equivalent to those on the 2005 Population Census of Japan [31]. A total of 7,501 potential respondents were randomly and blindly selected according to the set sample size and attributes from the registered samples and were subsequently invited via e-mail to participate in the Internet-based survey (response rate = 25.7%). The invitation e-mail contained a URL directing the potential respondents to a protected area of the Web site where the questionnaire was located. They could then log on using their own login ID and password. As an incentive for participation, the Internet research service organization offered reward points valued at 40 yen. One U.S. dollar was equivalent to approximately 109 yen at the time. All the respondents voluntarily completed a demographic data information form and clicked on the "agree" button at the end of an online informed consent form approved by the Institutional Review Board.

### Measurements

#### Physical activity

The short version of the IPAQ was utilized to estimate the amount of physical activity the participants engaged in. The IPAQ was designed for adults 18–65 years of age for the purpose of identifying the frequency and duration of walking, moderate and vigorous physical activity, and sedentary activity during the past week [32]. The one-week test-retest reliability of the short, self-administered, Japanese version of the IPAQ is good (Spearman's ρ = .72–.93), and the criterion validity for the Japanese version of the IPAQ against an accelerometer is acceptable (Spearman's ρ = .39) [33].

The short form data were utilized to estimate the total weekly physical activity level (METs·hour per week) by weighting the reported hours per week within each of the three activity categories–walking, moderate activity, and vigorous activity–by MET energy expenditure estimates assigned to each activity category. The METs value for each category was obtained from the study of Craig et al. [32]. The Japanese guidelines recommend 23 METs·hour per week of physical activity at an intensity level of at least 3 METs [8]. A dichotomous physical activity variable was created by defining the physical activity level of those who reported engaging in 23 METs·hour or more per week of physical activity as "recommended" and that of those who reported less than 23 METs·hour per week of physical activity as "not recommended."

#### Demographic variables

Demographic variables included gender, age, marital status, educational level, household income, and employment status. Age was classified in years as 20–29, 30–39, 40–49, and 50 or older. Marital status was categorized as currently married or currently unmarried. Educational level was classified as less than high school graduate, junior college graduate or equivalent, and college graduate or higher. Household income was classified into five categories, ranging from less than 3,000,000 yen to 10,000,000 yen or more annually. Employment status was categorized as employed or not employed.

#### Psychological variables

Self-efficacy for exercise was measured using a four-item scale that assessed the confidence of participants in engaging in a physical activity when faced with common barriers [34]. The participants were asked to rate their level of confidence using a 5-point Likert scale ranging from 1 (strongly disagree) to 5 (strongly agree) in terms of their ability to be physically active under the following conditions: physical tiredness, poor weather conditions, lack of time, and psychological stress. For this scale, the two-week test-retest reliability (r = .78) and internal consistency (α = .84) were found to be good [34]. All the items were subsequently summed to form a single self-efficacy variable, which was dichotomized into high and low categories based on a median split.

The perceived positive (pros) and negative aspects (cons) of exercise were assessed by a decisional balance measure, including a 10-item pros scale and a 10-item cons scale [35]. Examples of items in the pros scale were "Regular exercise would help me relieve tension" and "It would be easier for me to perform routine physical tasks if I exercised regularly." Examples of items in the cons scale were "Regular exercise would take too much of my time" and "I would have less time for my family and friends if I exercised regularly." The participants rated the extent to which they agreed or disagreed with each item on a 5-point Likert scale ranging from 1 (strongly disagree) to 5 (strongly agree); good two-week test-retest reliability (pros: r = .80; cons: r = .77) and internal consistency (pros and cons scales: α = .84) were reported [35]. All the items were subsequently summed to form a single item for pros and cons and dichotomized into high and low categories using a median split.

#### Social variables

Social support for exercise from family and friends was measured using a five-item scale [25,36,37]. This scale assessed functional, emotional, and informational social support for exercise using a 5-point Likert scale that measured the agreement or disagreement of participants on a scale of 1 (strongly disagree) to 5 (strongly agree) for the following five aspects: advice/instruction, understanding/sympathy, encouragement/reinforcement, joint implementation, and compliment/appreciation. This scale was found to have good internal consistency (α = .86). In addition, confirmatory factor analysis was conducted in order to examine the construct validity for this scale; the analysis revealed that five items were loaded consistently with one construct. Data indicated good model fits (GFI = 0.98; AGFI = 93; CFI = 1.00; RMSEA = 0.07) [36]. Additionally, social support scores were observed to be associated with the stage of exercise adoption (p < .001) [36]. All the items were subsequently summed to form a single item for social support and dichotomized into high and low categories using a median split.

By referring to previous research [38], a question regarding professional advice/support was asked: "Within the last year, have you been advised by a physician and/or health professional to exercise more?" The participants responded by answering yes or no.

#### Environmental variables

The perceived neighborhood environments for physical activity, which may act as facilitators or barriers to physical activity, were assessed by five items obtained from the previous investigation [25]. These five items are as follows: (1) I possess home fitness equipment (shoes, pedometer, dumbbells, etc.), (2) My neighborhood provides facilities (walking trail, park, fitness club, etc.) for engaging in physical activity, (3) My neighborhood provides a safe and well-maintained environment (adequate lighting and sidewalks, light traffic volume, etc.) for being physically active, (4) I have access to enjoyable scenery when engaging in physical activity, and (5) I frequently observe other people exercising. Each item was assessed on a 4-point Likert scale ranging from 1 (strongly disagree) to 4 (strongly agree). Responses were dichotomized into two categories: yes (strongly agree or agree) and no (strongly disagree or disagree). Furthermore, the perceived (self-reported) residence area was obtained by asking participants to state whether their residential area was urban, suburban, or rural.

### Statistical Analyses

The data were analyzed for 1,932 adults who provided complete information for the study variables. All the analyses were stratified by gender. Initial analyses were conducted with unadjusted logistic regression to examine whether or not each demographic, psychological, social, and environmental variable was related to meeting physical activity recommendations (recommended vs. not recommended). Subsequently, forced-entry adjusted logistic regression analyses were conducted to examine the association of the demographic, psychological, social, and environmental variables with attaining the recommended level of physical activity. Unadjusted and adjusted odd ratios (ORs) and 95% confidence intervals (CI) were calculated for each variable.

The independent variables included gender, age, marital status, educational status, employment status, household income, self-efficacy, pros, cons, social support, advice from a health professional, home fitness equipment, access to facilities, neighborhood safety, enjoyable scenery, frequently observing others exercising, and urban location. The statistical significance was considered to be p < .05. The Statistical Package for Social Science (SPSS) for Windows 14.0 was utilized to compute the statistics [39].

## Results

### Basic characteristics of the respondents

In the present study, 962 men and 970 women were classified into two groups according to their self-reported physical activity level. Table 1 presents the demographic characteristics of the respondents and comparative data from the 2005 and 2000 Population Census of Japan and the 2006 National Livelihood Survey [31,40,41]. The frequencies for gender, age, marital status, and employment status in the respondents were relatively similar to those in the census data. However, the respondents had higher educational status and household income as compared to the general Japanese population.

**Table 1:** Basic characteristics of respondents

	Participants						
		
	Men	Women		Total		*^#^General Japanese	
	n	%	N	%	n	%	%
**Total**	962	49.8	970	50.2	1932	100.0	-
**Age**							
20–29	192	20.0	195	20.1	387	20.0	15.9
30–39	196	20.4	191	19.7	387	20.0	19.0
40–49	197	20.5	196	20.2	393	20.3	16.3
≥50	377	39.2	388	40.0	765	39.6	48.8
**Marital status**							
Married	606	63.0	703	72.5	1309	67.8	64.5
Unmarried	356	37.0	267	27.5	623	32.2	35.5
**Educational status**							
≤ high school graduate	237	24.6	292	30.1	529	27.4	67.7
2 years of college	125	13.0	358	36.9	483	25.0	12.6
≥ college graduate	600	62.4	320	33.0	920	47.6	15.5
**Employment status**							
Employed	801	83.3	496	51.1	1297	67.1	56.0
Not employed	161	16.7	474	48.9	635	32.9	44.0
**Household income**							
< 3,000,000 yen	150	15.6	160	16.5	310	16.0	30.6
3,000,000–yen	283	29.4	265	27.3	548	28.4	23.2
5,000,000–yen	201	20.9	195	20.1	396	20.5	13.5
7,000,000–yen	202	21.0	214	22.1	416	21.5	17.2
10,000,000–yen	126	13.1	136	14.0	262	13.6	11.3
No	658	68.4	817	84.2	1475	76.3	-
Yes	304	31.6	153	23.7	457	23.7	-

Study conducted by Internet research service organization, Tokyo, Japan, in February 2008.

*Reference: Age, Marital status, and Employed status: 2005 Population Census of Japan; Educational status: 2000 Population Census of Japan; Household income levels: 2006 National Livelihood Survey

# Data of all those aged 19 and below were excluded.

### Association between demographic variables and attaining the recommended level of physical activity

Overall, 28.3% of the respondents attained the recommended level of physical activity; however, men (31.6%) surpassed women (24.9%) in this regard (χ^2^(1) = 10.5, p = 0.001). For men, there were no significant associations between any demographic variables and meeting the recommended level of physical activity (Table 2). However, unmarried women were significantly less likely to be physically active at the recommended level than married women (OR = 0.65; 95% CI: 0.46–0.92). On the other hand, those who were junior college graduates or equivalent were significantly more likely to attain the recommended level of physical activity than those who were high school graduates or less (OR = 1.45; 95% CI: 1.01–2.08). Moreover, those with the highest level of household income were significantly more likely to be physically active at the recommended level than those with the lowest level of household income (OR = 0.65; 95% CI: 0.46–0.91).

**Table 2:** Associations between sociodemographic variables and meeting physical activity recommendations

	Meeting physical activity recommendations					
	
	Men (n = 962)			Women (n = 970)		
		
	%	unadjusted OR	(95% CI)	%	unadjusted OR	(95% CI)
**Total**	31.6			24.9		
**Age**						
20–29	30.7	1.00	(ref)	22.1	1.00	(ref)
30–39	30.6	0.99	(0.65–1.53)	18.9	0.82	(0.50–1.35)
40–49	30.5	0.99	(0.64–1.52)	25.5	1.21	(0.76–1.93)
≥50	33.2	1.12	(0.77–1.63)	29.1	1.45	(0.97–2.17)
**Marital status**						
Married	32.5	1.00	(ref)	27.0	1.00	(ref)
Unmarried	30.1	0.89	(0.67–1.18)	19.5	0.65	(0.46–0.92)
**Educational status**						
≤ high school graduate	34.2	1.00	(ref)	21.6	1.00	(ref)
2 years of college	40.8	1.33	(0.85–2.07)	28.5	1.45	(1.01–2.08)
≥ college graduate	28.7	0.77	(0.56–1.07)	24.1	1.15	(0.79–1.68)
**Employment status**						
Employed	30.6	1.00	(ref)	23.6	1.00	(ref)
Not employed	36.6	1.31	(0.92–1.87)	26.4	1.16	(0.87–1.55)
**Household income**						
< 3,000,000 yen	33.3	1.00	(ref)	21.9	1.00	(ref)
3,000,000–yen	29.7	0.84	(0.55–1.29)	21.1	0.96	(0.59–1.54)
5,000,000–yen	30.3	0.87	(0.55–1.37)	28.2	1.40	(0.86–2.28)
7,000,000–yen	34.2	1.04	(0.66–1.62)	23.4	1.09	(0.67–1.78)
10,000,000–yen	29.7	0.93	(0.56–1.54)	28.6	1.83	(1.09–3.06)

Study conducted by Internet research service organization, Tokyo, Japan, in February 2008.

% = the prevalence of meeting physical activity recommendation

*p < .05

### Univariate association between attaining the recommended level of physical activity and psychological, social, and environmental variables

Table 3 presents the univariate relationship between attaining the recommended level of physical activity and psychological, social, and environmental factors by gender. A majority of the psychological, social, and environmental factors were significantly associated with attaining the recommended level of physical activity. For both men and women, those who reported a high level of self-efficacy; greater pros and fewer cons; a high level of social support; possessing home fitness equipment; access to facilities, neighborhood safety, and enjoyable scenery; and frequently observing others exercising were significantly associated with meeting the physical activity recommendations. For both genders, advice from a health professional and residential area were not significantly associated with attaining the recommended level of physical activity.

**Table 3:** Unadjusted odds ratios for meeting physical activity recommendations among Japanese adults

	Meeting physical activity recommendations					
	
	Men (n = 962)			Women (n = 970)		
	
	Unadjusted OR	(95% CI)		Unadjusted OR	(95% CI)	
**Self-efficacy**						
Low	1.00	(ref)		1.00	(ref)	
High	2.81	(2.10–3.76)	***	3.53	(3.23–8.42)	***
**Pros**						
Low	1.00	(ref)		1.00	(ref)	
High	1.86	(1.41–2.47)	***	1.93	(1.43–2.61)	***
**Cons**						
Low	1.00	(ref)		1.00	(ref)	
High	0.56	(0.43–0.75)	***	0.34	(0.25–0.47)	***
**Social support**						
Low	1.00	(ref)		1.00	(ref)	
High	2.04	(1.54–2.70)	***	1.88	(1.40–2.54)	***
**Health professional advice**						
No	1.00	(ref)		1.00	(ref)	
Yes	0.87	(0.65–1.17)		0.87	(0.58–1.32)	
**Home fitness equipment**						
No	1.00	(ref)		1.00	(ref)	
Yes	1.99	(1.51–2.62)	***	2.07	(1.54–2.79)	***
**Access to facilities**						
No	1.00	(ref)		1.00	(ref)	
Yes	1.59	(1.20–2.10)	**	1.91	(1.39–2.61)	***
**Neighborhood safety**						
No	1.00	(ref)		1.00	(ref)	
Yes	1.60	(1.21–2.11)	**	1.58	(1.17–2.12)	*
**Enjoyable scenery**						
No	1.00	(ref)		1.00	(ref)	
Yes	1.46	(1.11–1.91)	*	1.89	(1.40–2.55)	***
**Frequently observing others exercising**						
No	1.00	(ref)		1.00	(ref)	
Yes	1.52	(1.16–2.00)	*	1.55	(1.15–2.09)	*
**Residential area**						
Urban	1.00	(ref)		1.00	(ref)	
Suburban	1.08	(0.81–1.43)		1.08	(0.79–1.46)	
Rural	0.76	(0.44–1.31)		0.54	(0.29–1.00)	

OR = odd ratios; CI = confidence interval; ref = referent group

Study conducted by Internet research service organization, Tokyo, Japan, in February 2008.

*** p < .000, ** p < .01, *p < .05

### Multivariate association between attaining the recommended level of physical activity and psychological, social, and environmental variables

Table 4 presents the results of the adjusted logistic regression analysis. For men, self-efficacy (OR = 2.13; 95% CI: 1.55–2.94) was the strongest positive association with attaining the recommended level of physical activity followed by possessing home fitness equipment (OR = 1.55; 95% CI: 1.14–2.10) and social support (OR = 1.44; 95% CI: 1.06–1.97). For women, self-efficacy was also the strongest positive association with attaining the recommended level of physical activity (OR = 2.72; 95% CI: 1.82–4.08). Being a junior college graduate (OR = 1.54; 95% CI: 1.04–2.29), possessing home fitness equipment (OR = 1.41; 95% CI: 1.01–1.99), and reporting enjoyable scenery (OR = 1.60; 95% CI: 1.09–2.36) were positively associated with attaining the recommended level of physical activity, while reporting more cons to being active was inversely associated with the likelihood of meeting physical activity recommendations (OR = 0.47; 95% CI: 0.33–0.67). Women living in rural areas were half as likely as those living in urban areas to attain the recommended level of physical activity (OR = 0.50; 95% CI: 0.25–0.97).

**Table 4:** Adjusted odds ratios for meeting physical activity recommendations among Japanese adults

	Meeting physical activity recommendations					
	
	Men			Women		
	
	Adjusted OR	(95% CI)		Adjusted OR	(95% CI)	
**Age**						
20–29	1.00	(ref)		1.00	(ref)	
30–39	0.99	(0.60–1.64)		0.69	(0.40–1.21)	
40–49	0.93	(0.54–1.60)		0.97	(0.56–1.67)	
≥50	0.92	(0.55–1.54)		1.00	(0.60–1.67)	
**Marital status**						
Married	1.00	(ref)		1.00	(ref)	
Unmarried	0.95	(0.64–1.39)		0.83	(0.52–1.32)	
**Educational status**						
≤ high school graduate	1.00	(ref)		1.00	(ref)	
2 years of college	1.28	(0.79–2.07)		1.54	(1.04–2.29)	*
≥ college graduate	0.67	(0.47–0.95)		1.20	(0.79–1.84)	
**Employment status**						
Employed	1.00	(ref)		1.00	(ref)	
Not employed	1.11	(0.73–1.68)		0.95	(0.67–1.33)	
**Household income level**						
< 3,000,000 yen	1.00	(ref)		1.00	(ref)	
3,000,000–yen	0.79	(0.50–1.27)		0.86	(0.51–1.44)	
5,000,000–yen	0.82	(0.49–1.37)		1.13	(0.66–1.96)	
7,000,000–yen	0.92	(0.54–1.57)		0.67	(0.38–1.17)	
10,000,000–yen	0.77	(0.43–1.39)		1.16	(0.65–2.08)	
**Self-efficacy**						
Low	1.00	(ref)		1.00	(ref)	
High	2.13	(1.55–2.94)	***	2.72	(1.82–4.08)	***
**Pros**						
Low	1.00	(ref)		1.00	(ref)	
High	1.27	(0.92–1.75)		1.02	(0.72–1.46)	
**Cons**						
Low	1.00	(ref)		1.00	(ref)	
High	0.83	(0.60–1.14)		0.47	(0.33–0.67)	***
**Social support**						
Low	1.00	(ref)		1.00	(ref)	
High	1.44	(1.06–1.97)	*	1.13	(0.80–1.59)	
**Health professional advice**						
No	1	(ref)		1	(ref)	
Yes	0.89	(0.64–1.24)		0.66	(0.42–1.05)	
**Home fitness equipment**						
No	1.00	(ref)		1.00	(ref)	
Yes	1.55	(1.14–2.10)	*	1.41	(1.01–1.99)	*
**Access to facilities**						
No	1.00	(ref)		1.00	(ref)	
Yes	1.11	(0.79–1.56)		1.20	(0.82–1.77)	
**Neighborhood safety**						
No	1.00	(ref)		1.00	(ref)	
Yes	1.09	(0.76–1.56)		0.86	(0.58–1.27)	
**Enjoyable scenery**						
No	1.00	(ref)		1.00	(ref)	
Yes	1.03	(0.74–1.45)		1.60	(1.09–2.36)	*
**Frequently observing others exercising**						
No	1.00	(ref)		1.00	(ref)	
Yes	1.12	(0.79–1.57)		1.09	(0.75–1.58)	
**Residential area**						
Urban	1.00	(ref)		1.00	(ref)	
Suburban	1.03	(0.76–1.39)		1.04	(0.74–1.46)	
Rural	0.66	(0.36–1.21)		0.50	(0.25–0.97)	*

OR = odd ratios; CI = confidence interval; ref = referent group

Study conducted by Internet research service organization, Tokyo, Japan, in February 2008.

*** p < .000, ** p < .01, *p < .05

## Discussion

The present investigation examined the associations of psychological, social, and environmental factors with engagement in the recommended physical activity level among Japanese adults and determined that significant correlates emerged from all of these domains, and that there were some variations in association by gender. Although many of the psychological, social, and environmental factors, except health professional advice and residential area, were significantly related to attaining the recommended level of physical activity in the univariate analysis, a large number of these factors were no longer significant when incorporated into the multivariate analysis.

With regard to the psychological factors, the results of the current study replicated the findings of previous research in terms of self-efficacy, which is well-documented as a strong and consistent indicator of physical activity behavior [15,17,20,21,23,24]. The perceived positive (pros) and negative (cons) aspects of exercise were inconsistently associated in men and women. In the present study, only for women, beliefs regarding unfavorable features (cons) emerged as having a negative association with attaining the recommended level of physical activity. Lacking time and energy and engaging in daily activities were most commonly reported as barriers to regular physical activity among women [22,42,43], which may be a reflection of their overlapping obligations such as work, housekeeping, motherhood, and personal relationships. Previous research in other countries has found pros and cons to be a significant correlate of physical activity [24,29]. De Bourdeaudhuij and Sallis [29] examined the relative contribution of psychosocial variables among men and women in middle and old age groups. In their study, perceived benefits contributed to engagement in moderate to vigorous physical activity only in middle-aged men, whereas perceived barriers emerged as a negative correlate only in older women, thereby leading to the conclusion that the contribution of perceived benefits and barriers may depend on age and gender [29]. These findings imply that in terms of engaging in physical activity, women may be more vulnerable to barriers than men. Therefore, employing strategies to eliminate such barriers may enhance the effectiveness of future interventions in attaining the recommended physical activity level for women.

A positive association of social support with attaining the recommended level of physical activity has been previously found in numerous multivariate studies [16,17,21,23]. However, the current study revealed an association between social support and attaining the recommended level of physical activity only in men. Previous studies have examined the associations of social support on meeting the physical activity recommendation for men and women, and the results have been inconsistent [24,30,42]. Two studies reported a positive association only for women [30,42], whereas others reported no association for either men or women [24]. However, the "sufficiently active" physical activity level and the physical activity type that were the focus in the present study were defined differently from those in all three previous studies, which could be a reason for the contrasting results [24,30,42]. Moreover, certain studies have reported that social support has not a direct but rather an indirect effect on engaging in physical activity through self-efficacy and self-motivation [44,45]. Further detailed examinations of the direct and indirect influences of psychological, social, and environmental factors on meeting the recommended level of physical activity using structural equation analysis may clarify the role of social support for Japanese women.

Among the rapidly increasing number of international research studies that investigate associations between the physical environment and physical activity, it appears as though few studies have examined these associations among Japanese adults. Reviews of the literature regarding the environmental correlates of physical activity have concluded that there is weak or mixed evidence of associations between environmental factors and physical activity [11,46]. One review reported enjoyable scenery as a relatively weak positive correlate of physical activity [11]. Nevertheless, the findings of the present study are consistent with other previous studies that examined the relative association of psychosocial and environmental factors with engagement in physical activity for women, thereby suggesting that green space or beautiful landscapes may be an important environmental factor for motivating women to indulge in physical activity [22,42].

The current study also found that female residents in rural areas were the least likely to attain the recommended level of physical activity, which is consistent with previous studies [47,48]. Wilcox et al. [42] found that compared with urban women, rural women in the U.S. experienced more frequent perceived barriers to leisure-time physical activity, which may explain why rural women in the current study reported being less active than urban women. Further research on physical activity correlates among women living in rural areas is warranted. The positive association between possessing home fitness equipment and attaining the recommended level of physical activity in the case of both men and women is somewhat consistent with previous research [11]. Even though a previous study has reported a non-significant association between the two abovementioned factors, it examined only older Australian adults [21]. This finding implies that possessing home fitness equipment such as a pedometer and dumbbells may be an important environmental correlate of meeting the recommended level of physical activity among the Japanese population. However, being cross-sectional it may be that those who are more active are more likely to purchase and keep physical activity at home. Longitudinal research is needed to elucidate the direction of some of these relationships.

The current study found that neighborhood safety (adequate lighting and sidewalks, light traffic volume in physical activity participation, etc.) contributed very little toward promoting the recommended level of physical activity. Similarly, numerous previous studies reported a non-significant association, although the results were inconsistent [15,19-23,28,47,49-52]. As a possible explanation for the result in the present study, engaging in physical activity at the recommended level may affect an individual's perception of the environment (i.e., those who are more active are more likely to be aware of neighborhood safety issues). The limitations of measurement in the present study may be another reason for the lack of association. In the present study, the aspect of "neighborhood safety" was derived by asking participants to consider multiple indicators (lighting, sidewalk, and traffic volume). Brownson et al. [49] reported that heavy traffic and the existence of sidewalks were significantly associated with meeting physical activity recommendations, while streetlights were not. Thus, individual measurement of each safety indicator may be required in order to increase the sensitivity of these indicators.

The present study also failed to identify associations between access to facilities for engaging in physical activity and frequently observing others exercising and meeting the recommended level of physically activity, which are recognized as relatively consistent correlates of engagement in physical activity [11,14,17,21,22,28,42,49,51,53]. These perceived environmental factors may be mainly related to recreational or leisure-time physical activity [22,42,49,50,53]. Thus, utilizing the IPAQ, which does not assess the specific purposes of physical activity (e.g., physical activity for recreation, transportation, and occupation), may lead to an underestimation of the impact of these environmental factors on attaining the recommended level of physical activity in the present study [16,52,54].

In addition, the lack of association in the present study between access to facilities am being physically active may be attributed to ambiguous wording or mixed components in the question. The item that was utilized in the present study, "There are facilities (walking trail, park, and fitness club, etc.) to engage in physical activity in my neighborhood", could include both the accessibility and availability of the facilities. Certain previous studies have reported that accessibility, convenience, and safety of the facilities were probably more strongly associated with engaging in physical activity than availability [24,53]. Thus, making the measurement more sophisticated (e.g., more careful wording for the specific components of facilities in the question) may be necessary for future investigation.

Finally, the amount of physical activity recommended by the Japanese physical activity guideline, 23 METs·hour per week, may be a potential reason for the difference in the findings observed in the present study from those of studies conducted in other Western countries [8]. Approximately 60 minutes of physical activity at an intensity of 3.3 METs (e.g. walking), seven days per week, 420 minutes per week is equivalent to the recommended level in Japan; this is almost two to three times higher than other international public health guidelines on physical activity–at least 30 minutes of moderate-intensity physical activity five or more days per week [1,3-5]. Numerous previous studies conducted for examining the psychological, social, and environmental correlates of physical activity among adult populations utilized the international public guideline (e.g., 150–210 minutes and 10–14 METs·hour/week) as the physical activity criteria [14-18,21,23,24,50-52].

The current investigation had a number of limitations. First, the analysis was cross-sectional, thereby making it impossible to determine cause and effect. Next, the level of physical activity and all the psychological, social, and environmental variables were administered using only a self-reported questionnaire, which could be subject to bias. For example, Ishikawa-Takata et al. [55] found that only 36% of 150 healthy, free-living Japanese adults could be classified into the same level of physical activity groups (insufficiently active, sufficiently active, and highly active) according to both the total METs assessed by the IPAQ and physical activity level measured by the doubly labeled water method. Thus, recall bias and an inaccurate estimation of the attained physical activity level are unavoidable. Moreover, the present study assessed total physical activity using only the IPAQ. The IPAQ incorporates four domains of physical activity: domestic, occupational, transport, and recreational; however, neighborhood environmental factors were relevant to only two purposes: transport and recreational. Thus, a mismatch between the outcome and exposure was inevitable. Future research should match environmental influences with physical activity domain.

Finally, the current study was conducted in an Internet setting. Eysenbach et al. [56] indicated that issues of generalizability, mainly due to the selection bias, were important considerations because of the non-representative nature of the Internet population and the self-selection of participants for the survey. Rhodes et al. [57] suggest that younger, more educated, and higher-earning individuals have greater access to the Internet. In addition, people are more likely to respond to a survey if they are interested in its contents or are attracted by the incentives offered for participation [56-58]. Therefore, the basic characteristics of the respondents could be biased, which implies that the findings in such a setting may not be adequately applicable to the general population. In particular, a comparison of sample profiles with census data indicated that there was an overrepresentation of university-educated individuals, which suggests that the results may be less applicable to those who have received less education.

## Conclusion

In summary, significant correlates of attaining the recommended level of physical activity were observed in all the psychological, social, and environmental domains. In addition, men and women had different patterns of psychological, social, and environmental correlates. The findings of the current study contribute additional evidence to the literature on the multivariate factors associated with physical activity behavior and provide useful information for the development of interventions to increase the number of Japanese adults who meet physical activity recommendations. Future studies should match behavior-specific psychological, social, and environmental variables concomitantly with specific domains of physical activity, such as walking for transport or recreational physical activity. Rural women should be a particular focus of future studies of the Japanese population. Finally, promoting self-efficacy for engaging in physical activity, utilizing home fitness equipment, locating or enhancing green spaces or beautiful landscapes in a neighborhood, and promoting social support for men as well as eliminating the barriers for women may be effective strategies for future interventions aimed at increasing physical activity levels to those recommended for Japanese adults.

## Competing interests

The authors declare that they have no competing interests.

## Authors' contributions

**AS **participated in the design of the study, performed the statistical analyses, and drafted the manuscript. **YN **and **IM **performed the sequence alignment and helped in drafting the manuscript. **OK **conceived the study, participated in its design and coordination, and helped in drafting the manuscript. **HK **participated in the design of the study and helped in data processing. All the authors have read and approved the final manuscript.
